# Asiatic Acid Attenuates Myocardial Ischemia/Reperfusion Injury via Akt/GSK-3β/HIF-1α Signaling in Rat H9c2 Cardiomyocytes

**DOI:** 10.3390/molecules21091248

**Published:** 2016-09-19

**Authors:** Xiang Huang, Li Zuo, Yanni Lv, Chuqiao Chen, Yaqin Yang, Hongbo Xin, Yunman Li, Yisong Qian

**Affiliations:** 1 Institute of Translational Medicine, Nanchang University, 1299 Xuefu Avenue, Nanchang 330031, China; xiangh0608@gmail.com (X.H.); zuolnjau@163.com (L.Z.); chenchuqiaoccq@163.com (C.C.); m13870991340@163.com (Y.Y.); xinhb2009@sina.com (H.X.); 2 Department of Pharmacy, The First Affiliated Hospital of Nanchang University, 17 Yongwai Street, Nanchang 330006, China; lvyanni@126.com; 3 Department of Physiology, China Pharmaceutical University, 24 Tongjiaxiang, Nanjing 210009, China; phy.lym@gmail.com

**Keywords:** asiatic acid, myocardial ischemia, OGD/R, Akt, HIF-1α

## Abstract

Myocardial ischemic/reperfusion injury results from severe impairment of coronary blood supply and leads to irreversible cell death, with limited therapeutic possibilities. Asiatic acid is a pentacyclic triterpenoid derived from the tropical medicinal plant *Centella asiatica* and serves a variety of bioactivities. In this study, we determined the effect of asiatic acid on myocardial ischemia/reperfusion injury and investigated the underlying mechanisms, using an in vitro rat H9c2 cardiomyocytes model of oxygen-glucose deprivation/reoxygenation (OGD/R) injury. Results showed that pre-treatment with asiatic acid significantly augmented cell viability and prevented lactate dehydrogenase (LDH) release in a concentration-dependent manner after OGD/R exposure. Asiatic acid at 10 μM effectively inhibited apoptotic cell death, suppressed the activities of caspase-3 and caspase-9, and reversed Bax/Bcl-2 ratio in hypoxic H9c2 cells. In addition, asiatic acid improved mitochondrial function, as evidenced by reduced reactive oxygen species (ROS) accumulation, enhanced mitochondrial membrane potential and decreased intracellular calcium concentration. Using Western blot assay, we found that asiatic acid promoted the phosphorylation of Akt and subsequent inactivation of glycogen synthase kinase-3β (GSK-3β), and induced the expression of hypoxia-inducible factor 1α (HIF-1α) after OGD/R. The cardioprotective effects of asiatic acid were attenuated by the Akt or HIF-1α inhibitor. Taken together, these data suggested that asiatic acid exerted protective effects against OGD/R-induced apoptosis in cardiomyocytes, at least partly via the Akt/GSK-3β/HIF-1α pathway.

## 1. Introduction

Myocardial ischemia injury followed by prolonged reperfusion results from an imbalance between myocardial oxygen supply and demand [1]. During ischemia, ATP depletion impairs the Ca^2+^ uptake capacity of the sarcoplasmic reticulum, leading to mitochondrial Ca^2+^ accumulation. Reintroduction of oxygen during reperfusion causes damage to the electron transport chain and results in increased mitochondrial generation of reactive oxygen species (ROS) [2]. Both mitochondrial Ca^2+^ overload and increased ROS contribute to the opening of the mitochondrial permeability transition pore, which further disturbs cellular energetics and triggers the irreversible apoptotic and necrotic cell death [3,4]. Recently, intense investigation has produced considerable insight into the pathobiology of myocardial ischemic injury, and the pharmacological intervention for cardioprotection may offer novel therapeutic approaches to ameliorate the risk and progression for heart diseases. However, the detailed mechanisms of myocardial ischemia injury remain to be elucidated.

**Figure 1:**
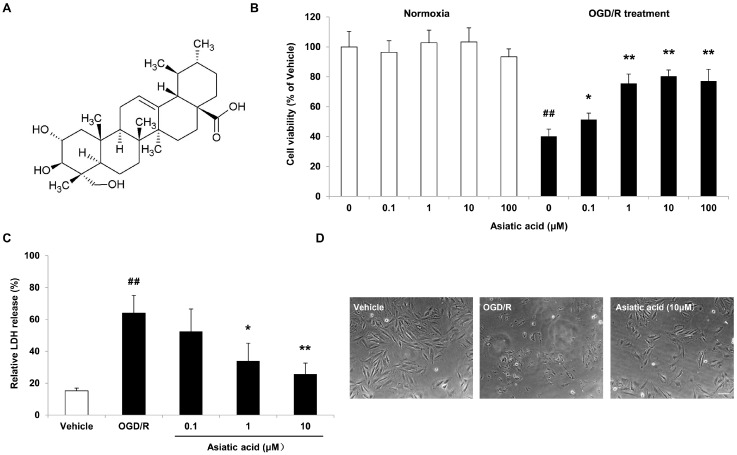
Asiatic acid attenuates oxygen-glucose deprivation/reoxygenation (OGD/R)-induced cell death in H9c2 rat cardiomyocytes. (**A**) Chemical structure of asiatic acid; (**B**) The MTT (3-(4,5-dimethylthiazol-2-yl)-2,5-diphenyltetrazolium bromide) tetrazolium reduction assay for cell viability under normoxia or subjected to OGD/R; (**C**) Determination of the relative LDH release after OGD/R exposure; (**D**) Morphology alterations after OGD/R injury under an inverted phase microscope. ## *p* < 0.01 vs. the vehicle group; * *p* < 0.05, ** *p* < 0.01 vs. the OGD/R group. Scale bar, 100 μm.

Asiatic acid (Figure 1A), a natural pentacyclic triterpene derived from *Centella asiatica* (Apiaceae family), elicits multiple bioactivities, including antioxidative, anti-inflammatory, anti-apoptotic and anti-glycative effects [5,6,7,8,9]. Recently, several studies have provided evidence that asiatic acid exerts beneficial effects against ischemic injury. Asiatic acid has been shown to inhibit left ventricular remodeling and improve cardiac function by blocking the phosphorylation of p38 mitogen-activated protein kinase (MAPK) and extracellular signal-regulated kinase 1/2 (ERK1/2) in the infarct border zone of the ischemic myocardium [10]. In focal ischemia models, asiatic acid attenuates infarct volume and improves mitochondrial dysfunction by inhibiting matrix metalloproteinase-9 induction and activation [11,12]. Moreover, asiatic acid has been implicated in neuroprotection in multiple in vitro models. Pre-treatment with asiatic acid attenuates glutamate-induced cell death by promoting the expression of peroxisome proliferator-activated receptor gamma coactivator 1-alpha (PGC-1α) and Sirtuin 1 in SH-SY5Y human neuroblastoma cells [13]. Asiatic acid decreases ROS accumulation and prevents apoptosis in primary neurons by reducing the cytosolic release of HtrA2/Omi, the up-regulation of Bax and caspase-3, as well as the dephosphorylation of ERK1/2 following C_2_-ceramide treatment [14]. However the underlying signal transduction pathways mediated by asiatic acid following myocardial ischemia are largely unknown.

In the present study, we investigated the potential cellular and molecular mechanisms by which asiatic acid might counteract myocardial cell death, using a cell line of H9c2 rat cardiomyocytes exposed to oxygen-glucose deprivation/reoxygenation (OGD/R) as an in vitro cellular model associated with myocardial ischemia and reperfusion injury.

## 2. Results

### 2.1. Asiatic Acid Attenuates OGD/R-Induced Cell Death in H9c2 Rat Cardiomyocytes

After 1 h of OGD followed by 24 h of reoxygenation, a significant decrease in cell viability occurred in H9c2 cells, with a 59.8% reduction of cell viability, as determined by the MTT (3-(4,5-dimethylthiazol-2-yl)-2,5-diphenyltetrazolium bromide) tetrazolium reduction assay. Asiatic acid pre-treatment at 0.1 to 10 μM for 4 h improved cell viability in a concentration-dependent manner. As the concentration was increased to 100 μM, asiatic acid did not show further protection. In addition, Asiatic acid with these concentrations did not affect cell viability under normoxia (Figure 1B). These results were further confirmed by lactate dehydrogenase (LDH) release assay. As expected, OGD/R induced an obvious increase of LDH release in H9c2 cells, while asiatic acid at 1 μM and 10 μM effectively prevented cell damage, with a reduction of 46.9% and 59.7% compared with the OGD/R group, respectively (Figure 1C). Moreover, morphological changes of H9c2 cells were assessed by light microscopic observations. As shown in Figure 1D, OGD/R elicited remarkable cell injury, which was significantly attenuated by asiatic acid treatment, as evidenced by decreased cell morphologic impairment.

### 2.2. Asiatic Acid Prevents Apoptosis in H9c2 Cardiomyocytes after OGD/R

**Figure 2:**
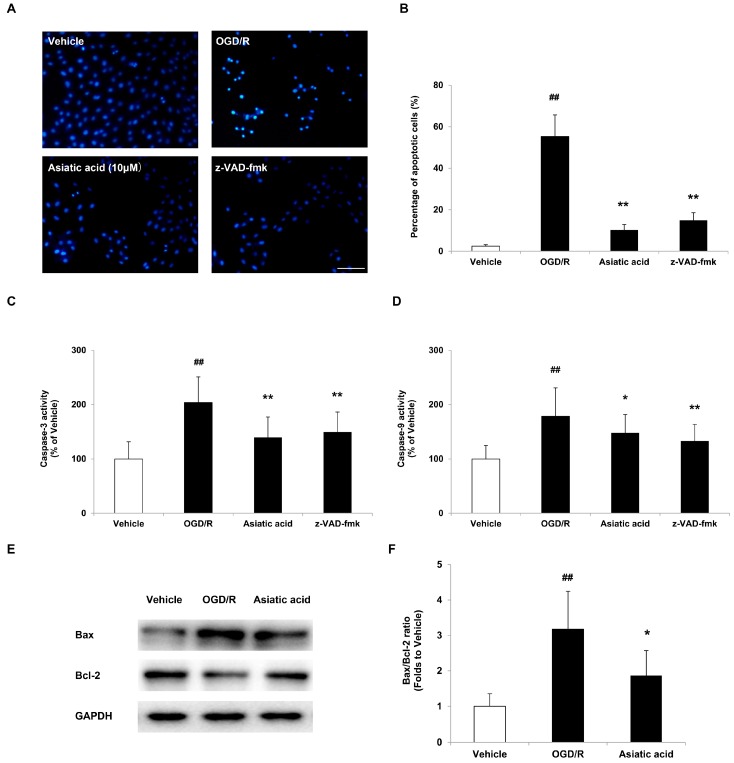
Asiatic acid attenuates apoptosis in H9c2 cardiomyocytes after oxygen-glucose deprivation/reoxygenation (OGD/R). (**A**) Nuclear condensation was visualized by Hoechst 33258 staining. Nuclei showing clearly bright chromatin condensation and/or fragmentation were regarded as apoptotic cells; (**B**) Apoptotic cells were counted and expressed as the percentage of total cell count; (**C**) Measurement of caspase-3 activity; (**D**) Measurement of caspase-9 activity; (**E**) Immunoblots of Bax, Bcl-2 and glyceraldehyde-3-phosphate dehydrogenase (GAPDH) by Western blot assay; (**F**) Semi-quantitative analysis of protein levels. ## *p* < 0.01 vs. the vehicle group; * *p* < 0.05, ** *p* < 0.01 vs. the OGD/R group. Scale bar, 100 μm.

According to the above result, the maximal protective effects were afforded by 10 μM asiatic acid. Therefore, we used 10 μM asiatic acid to further investigate its anti-apoptotic effects in H9c2 cardiomyocytes after OGD/R. Morphological determination of apoptosis was performed by labeling the cells with the nuclear stain Hoechst 33258 and visualization by fluorescence microscopy. The vehicle-treated cells showed intact nuclei and weak fluorescence whereas cells exposed to OGD/R displayed typical nuclear apoptotic morphology, as indicated by obvious nuclear fragmentation and condensation. However, pre-treatment with 10 μM asiatic acid resulted in a profound decrease in apoptotic cell death and an improvement in morphological properties (Figure 2A). The quantitative results showed that the percentage of apoptotic cells in the vehicle group was 2.39%, while the level of apoptotic H9c2 cells exposed to OGD/R increased to 52.9% compared with the basal level. Conversely, asiatic acid lowered the level of apoptotic cells to 7.73% after OGD/R insult (Figure 2B).

The activation of caspase-9 and caspase-3 is involved in the execution-phase of cell apoptosis and plays a central role in the mitochondria-dependent apoptotic pathway. Therefore, the effects of asiatic acid on caspase-3 and caspase-9 activities in H9c2 cells under OGD/R condition were examined. Following OGD/R injury, the activation of caspase-3 and caspase-9 was observed, with an increase of 103.76% and 78.46% compared with the vehicle group, respectively. Asiatic acid at 10 μM significantly attenuated these changes in H9c2 cells exposed to OGD/R (Figure 2C,D). By using the pan-caspase inhibitor, carbobenzoxy-valyl-alanyl-aspartyl-(O-methyl)-fluoromethylketone (z-VAD-fmk, 100 μM, for 1 h incubation), we observed a similar anti-apoptotic effect with decreased caspase activities (Figure 2A–D). These results indicated that the cardioprotective role of asiatic acid was partly associated to a regulation of caspase-dependent pathways.

Previous study has shown that the Bax/Bcl-2 ratio determines the apoptotic potential of a cell [15]. In our experiment, a marked increase in the Bax/Bcl-2 expression ratio was observed after OGD/R exposure by Western blot analysis. Treatment with 10 μM asiatic acid reduced the expression of Bax and enhanced that of Bcl-2, and thereby significantly decreased the Bax/Bcl-2 ratio in hypoxic H9c2 cells (Figure 2E,F).

### 2.3. Asiatic Acid Prevents Mitochondrial Dysfunction Following OGD/R Injury

**Figure 3:**
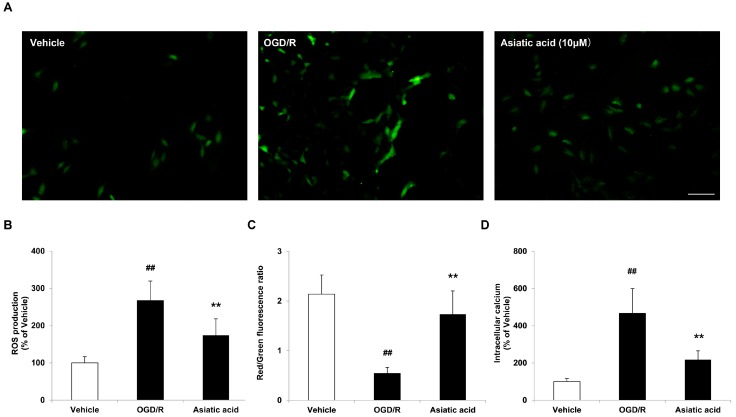
Asiatic acid prevents mitochondrial dysfunction following oxygen-glucose deprivation/reoxygenation (OGD/R) injury. (**A**) Reactive oxygen species (ROS) production as visualized by dichlorofluorescein (DCF) fluorescence; (**B**) Quantitative analysis of ROS production by measurement of fluorescence intensity with a microplate reader; (**C**) Measurement of mitochondrial membrane potential with JC-1-fluorescent dye; (**D**) Determination of intracellular calcium by Fluo-3 AM. ## *p* < 0.01 vs. the vehicle group; ** *p* < 0.01 vs. the OGD/R group. Scale bar, 100 μm.

The mitochondria have been implicated as central executioners of cell death. OGD/R induces mitochondria dysfunction mediated by oxidative stress and by ATP depletion [16,17], as evidenced by excessive ROS production, loss of mitochondrial membrane potential (MMP) and increases in cytosolic Ca^2+^ overload, proceeding cell apoptosis and necrosis [18]. Therefore, we investigated whether the anti-apoptotic effects of asiatic acid are associated with improved mitochondrial function following OGD/R injury. The levels of intracellular ROS, the concentration of intracellular Ca^2+^ as well as MMP in H9c2 cells were evaluated after OGD/R injury with or without asiatic acid treatment. Figure 3A illustrates fluorescent dichlorofluorescein (DCF) changes in response to OGD/R stimulation. In the vehicle group, there was only a small number of fluorescent cells in each visual field was observed. OGD/R treatment provoked an elevation of DCF fluorescence, with a 2.67-fold increase compared with the vehicle. In contrast, the number and intensity of fluorescent cells were markedly reduced in the asiatic acid-treated group; ROS generation was alleviated by 34.8% according to the quantitative result (Figure 3B). JC-1 was used to evaluate the loss of MMP in H9c2 cells. OGD/R led to a significant decrease in red/green fluorescence ratio, which was markedly reversed by asiatic acid (Figure 3C). In addition, the level of intracellular Ca^2+^ in H9c2 cells was markedly increased after OGD/R exposure and such augments were inhibited by asiatic acid treatment (Figure 3D). These results suggested that asiatic acid protected cardiomyocytes from apoptotic cell death partly by suppressing mitochondrial dysfunction.

### 2.4. Asiatic Acid Regulates the Akt/GSK-3β Signal Pathway and Increases HIF-1α Levels in Hypoxic H9c2 Cells

**Figure 4:**
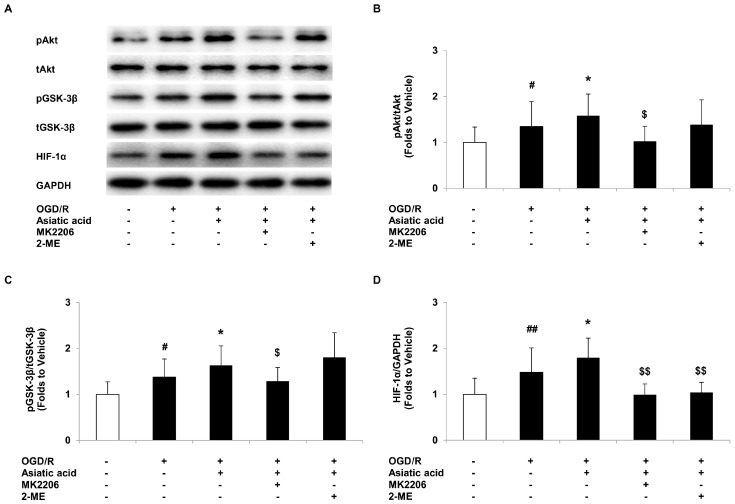
Asiatic acid regulates the Akt/GSK-3β signal pathway and increases hypoxia-inducible factor 1α (HIF-1α) levels in hypoxic H9c2 cells. (**A**) Immunoblots of phospho-Akt (pAkt), total Akt (tAkt), phospho-GSK-3β (pGSK-3β), total GSK-3β (tGSK-3β), HIF-1α and GAPDH; (**B**) Quantitative analysis of pAkt levels normalized to tAkt levels; (**C**) Quantitative analysis of pGSK-3β levels normalized to tGSK-3β levels; (**D**) Quantitative analysis of HIF-1α levels normalized to GAPDH levels. # *p* < 0.05, ## *p* < 0.01 vs. the vehicle group; * *p* < 0.05 vs. the OGD/R group; $ *p* < 0.05, $$ *p* < 0.01 vs. the asiatic acid-treated group.

To understand the molecular events for asiatic acid-mediated cardioprotection, Western blot analysis was performed to investigate the signal pathways involved in myocardial ischemia/reperfusion. The PI3-kinase/Akt activation leading to GSK-3β inactivation has been proposed to play a critical role in myocardial ischemia and compounds that have effects on this pathway may afford cardioprotection [19,20]. Our data revealed that OGD/R induced a 1.35-fold increase in Akt phosphorylation compared with the vehicle group, and asiatic acid further enhanced phospho-Akt levels in hypoxic H9c2 cells (Figure 4A,B). The inactivation of GSK-3β correlated with increased levels of phospho-Akt in our model, as evaluated by phosphorylation of GSK-3β at Ser9. Similarly, asiatic acid treatment caused profound elevation of GSK-3β phosphorylation, approximately 1.6 folds compared with the vehicle-treated group (Figure 4A,C).

HIF-1α is considered as the main regulator of angiogenesis and metabolism in response to hypoxia [21]. Recent studies showed that HIF-1α may provide a protective mechanism in cardiomyocytes under hypoxia [22,23]. Coincided with the previous studies, we demonstrated that OGD/R resulted in the up-regulation of HIF-1α, and asiatic acid further increased HIF-1α expression in hypoxic H9c2 cells (Figure 4A,D).

By using the Akt inhibitor MK2206, which was added into the cultures for 1 h incubation at 0.2 μM in combination with 10 μM asiatic acid, we found that the activation of the Akt/GSK-3β signaling pathway was blocked with the co-treatment, which reduced phospho-Akt and the phospho-GSK-3β levels compared with the asiatic acid-treated group. Whereas 2-ME, the HIF-1α inhibitor, combined with asiatic acid for 1 h incubation at 10 μM , had no effect on Akt/GSK-3β activation (Figure 4A–C). In addition, MK2206 or 2-ME co-treated with asiatic acid significantly inhibited HIF-1α expression after OGD/R when compared with the single treatment (Figure 4A,D). These results indicated that asiatic acid-mediated HIF-1α expression may be regulated by the Akt/GSK-3β pathway. 

### 2.5. Inhibition of Akt or HIF-1α Attenuates the Protective Effects of Asiatic Acid against OGD/R Injury

**Figure 5:**
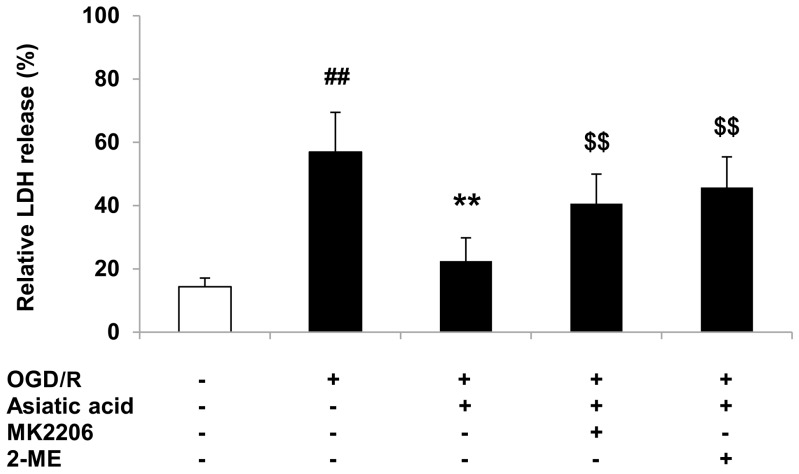
Inhibition of Akt or HIF-1α attenuates the protective effects of asiatic acid in OGD/R-injured H9c2 cells by LDH release assay. ## *p* < 0.01 vs. the vehicle group; ** *p* < 0.01 vs. the OGD/R group; $$ *p* < 0.01 vs. the asiatic acid-treated group.

In order to further determine the involvement of Akt/GSK-3β and HIF-1α in the cardioprotective effects of asiatic acid, the Akt inhibitor MK2206 and the HIF-1α inhibitor 2-ME were employed to evaluate the cell injury following OGD/R. The results showed that pre-treatment of H9c2 cells with MK2206 or 2-ME partly attenuated the protective activities of asiatic acid against hypoxia-induced cell death, as shown by increased LDH release (Figure 5). These data indicated that asiatic acid modulates Akt/GSK-3β and HIF-1α, which in turn, provide cardioprotection in H9c2 cells.

## 3. Discussion

Asiatic acid is a natural product with low toxicity, which is considered to be used as a dietary supplement for people at risk of cardiovascular diseases. Therefore, we used the pre-treatment protocol to observe its effects on the prevention of myocardial ischemia. The results demonstrated that asiatic acid effectively prevents apoptosis and improves mitochondrial function in H9c2 cells subjected to OGD/R. We found that the cardioprotective potency of asiatic acid was probably associated with the activation of the Akt/GSK-3β pathway and subsequent increase in HIF-1α levels in hypoxic cardiomyocytes. These findings suggest the possibility of asiatic acid intake as prevention strategies aimed at increasing post-ischemia outcomes. Cardiomyocytes cell death following acute myocardial infarction/reperfusion has been associated with cardiac dysfunction. It has been reported that myocardial apoptosis may attribute to cell death in ischemia/reperfusion injury [24]. Myocardial apoptosis is a complicated process that is mediated by a series of enzymes and molecules, including the opening of the mitochondrial permeability transition pore, release of cytochrome c and activation of caspases. Therefore, the factors that are involved in myocardial apoptosis were examined in our study. We found that asiatic acid significantly reduced the number of apoptotic cells, attenuated the activities of caspase-3 and caspase-9, and reversed the Bax/Bcl-2 ratio after OGD/R injury. These results revealed that asiatic acid has anti-apoptotic potency in cardiomyocytes.

Emerging evidence has suggested that mitochondria play a central role in regulating the apoptotic process [25]. Mitochondria have been recognized as a main source of ROS following ischemia/reperfusion injury [26]. Excessive amounts of ROS produced during reperfusion lead to damage of the antioxidant system, an increased membrane permeability, and subsequent calcium overload. The elevation of Ca^2+^ in the mitochondria could induce an opening of the mitochondrial permeability transition pore, exacerbate the loss of MMP and further stimulate ROS production. The interactions between ROS and Ca^2+^ produce a self-amplified, positive feed-back loop, which results in the exacerbation of apoptotic cellular damage during ischemic injury [27]. We observed in this study that asiatic acid dramatically suppressed ROS overproduction, prevented the dissipation of MMP and modulated intracellular calcium levels following OGD/R insults. Therefore, it can be speculated that asiatic acid exerts cardioprotective effects through regulating the ROS- Ca^2+^ feedback loop, thereby inhibiting mitochondria-mediated apoptotic cell death.

The molecular mechanism of cardioprotection provided by asiatic acid still needs to be elucidated, although several signal transduction pathways involved in the regulation of this compound are proposed in ischemic injury and other diseases. It has been revealed that asiatic acid induced Akt kinase activation in diabetic rats [28,29]. Since Akt functions to promote cardiomyocyte survival in ischemia/reperfusion injury [30], the effects of asiatic acid on the Akt signaling pathway was examined in our study. As expected, asiatic acid also promotes Akt activation, with decreased cell death after OGD/R exposure in H9c2 cardiomyocytes. By using the Akt inhibitor, we confirmed that asiatic acid mediates ischemic injury, at least in part, via increased Akt activation.

The activation of Akt leads to phosphorylation of the downstream effectors GSK-3β, which is considered as a key player in regulating mitochondrial signaling and cell apoptosis during myocardial ischemia [31,32,33]. The activity of GSK-3β is regulated by phosphorylation of two specific amino acid residues, serine 9 and tyrosine 216. Phosphorylation of serine 9 results in the inactivation of GSK-3β, which prevents cell apoptosis and confers cardioprotection [34,35]. Previous studies indicated that the Akt/GSK-3β signaling pathway may be an endogenous negative feedback regulator that forms a compensatory mechanism to limit apoptotic events in response to harmful stimuli [36]. This may explain the up-regulation of p-Akt and p-GSK-3β after OGD/R exposure observed in our experiment, which is probably due to the activation of an endogenous protective mechanism. Consistent with the results achieved by inhibition of GSK-3β [34,37], our results demonstrated that enhanced phosphorylation of GSK-3β with asiatic acid treatment may contribute to attenuated cell death during ischemia/reperfusion. However, decreased phosphorylation of GSK-3β in the presence of the Akt inhibitor led to the impairment of cell viability after OGD/R insults. These data further confirmed that the Akt/ GSK-3β signalling pathway participates in the mechanisms by which asiatic acid affects myocardial ischemic injury.

The activation of Akt/GSK-3β signaling has been linked to increased HIF-1α levels in many studies [38,39,40]. HIF-1α functions as a protective factor in response to hypoxia, is able to stimulate the transcription of multiple genes and promote cell survival during myocardial ischemic injury [41,42,43]. In addition, the activation of HIF-1α shifts oxidative phosphorylation toward glycolysis under hypoxic conditions, thus inhibiting the excessive accumulation of ROS [44]. It has been demonstrated that the activation of HIF-1α provides cardioprotection against ischemic/reperfusion injury by preventing the opening of mitochondrial permeability transition pore [45]. Another study showed that suppression of HIF-1α-frataxin signaling leads to decreased cell viability under hypoxic stress, which is associated with the accumulation of mitochondrial iron and increased ROS production [22]. Here, we demonstrated a further increase in HIF-1α levels in H9c2 cells with the treatment of asiatic acid after OGD/R exposure. Moreover, attenuated HIF-1α expression and increased LDH release in the presence of an Akt inhibitor in hypoxic H9c2 cells further validated the direct effects of Akt activation on HIF-1α levels. Therefore, we suggested that asiatic acid-induced activation of the Akt/GSK-3β/HIF-1α pathway may contribute to the suppression of ROS overproduction and mitochondrial dysfunction in ischemic/reperfusion injury. This might be a new mechanism of asiatic acid for the protective effects in cardiomyopathy. Moreover, the work on the efficiency of asiatic acid administered after OGD/R injury is ongoing in our laboratory and warrants further investigation, which will provide insights into potential clinical use of this compound.

## 4. Materials and Methods

### 4.1. Materials 

H9c2 cell lines were purchased from American Type Culture Collection (ATCC, CRL-1446) (Rockville, MD, USA). Asiatic acid (97% purity) and 2-Methyoxyestradiol (2-ME) were purchased from Sigma-Aldrich (St. Louis, MO, USA), and were prepared as a stock solution by dissolving in dimethyl sulfoxide (DMSO, 0.5%, final concentration). The anti-Bcl-2, anti-Bax, anti-Akt and anti-phospho-Akt antibodies were obtained from Santa Cruz Biotechnology, Inc. (Santa Cruz, CA, USA); the GSK-3β and phospho-GSK-3β (Ser9) antibodies were from Cell Signaling Technology, Inc., (Cell Signaling, Danvers, MA, USA), and the glyceraldehyde phosphate dehydrogenase (GAPDH) antibody was obtained from KangChen Bio-tech Inc., (Shanghai, China). MK2206 was obtained from Biovision (Milpitas, CA, USA), and z-VAD-fmk was from Beyotime Institute of Biotechnology (Haimen, China).

### 4.2. Oxygen-Glucose Deprivation and Cell Culture Treatment

H9c2 cells were cultured in Dulbecco’s modified essential medium (DMEM, Life Technologies, Shanghai, China) containing 10% fetal bovine serum (FBS, Life Technologies), 1% streptomycin (100 μg/mL) and 1% penicillin (100 U/mL) at pH 7.4 in a 5% CO_2_ incubator at 37 °C. H9c2 cells were exposed to oxygen-glucose deprivation as previously described, with some modifications [46]. After adhering overnight, The DMEM was replaced by glucose-free Earle’s balanced salt solution (EBSS, pH 7.4) bubbled with 95% N_2_ and 5% CO_2_. The cells were then placed in an anaerobic chamber containing a mixture of 95% N_2_ and 5% CO_2_ humidified at 37 °C for 1 h. Oxygen-glucose deprivation was terminated by replacing the anoxic medium with fresh DMEM, and returning to normoxia for an additional 24 h. Asiatic acid with varying concentrations (0.1, 1, 10 and 100 μM) was added to the cultures 4 h before oxygen-glucose deprivation. After 24 h of reoxygenation, cell viability was determined by MTT assay. Cytotoxicity was measured by lactate dehydrogenase (LDH) release into the culture medium, according to the following equation: LDH release = (LDH activity in the medium/total LDH activity) × 100%, using a commercially available kit (Jiancheng Bioengineering Institute, Nanjing, China).

### 4.3. Hoechst 33258 Staining

Hoechst 33258 staining was employed to evaluate the nuclear condensation and characteristic features of apoptotic cells. Cells were rinsed with phosphate-buffered saline (PBS, pH 7.4) three times and fixed with 4% paraformaldehyde for 30 min at room temperature, followed by incubation with 5 μM Hoechst 33258 at 37 °C for 20 min. Fluorescence images were examined under the fluorescence microscope. Total cells and damaged cells were counted and the percentage of apoptotic cells was calculated.

### 4.4. Measurement of the Activities of Caspase-3 and Caspase-9

Caspase-3 and caspase-9 activity was measured in lysates of cells using the CaspACE™ Assay System, Colorimetric (Promega, Madison, WI, USA) and Caspase-Glo^®^ 9 Assay kit (Promega), following the manufacturer’s instructions. Briefly, cells were lysed by freeze-thaw, and then incubated on ice for 20 min to ensure complete cell lysis. Cell lysates were centrifuged at 12,000 rpm for 10 min at 4 °C, and the supernatant fraction was collected for the determination. For caspase-3 activity assay, an aliquot of culture supernatant was incubated with 200 μM of DEVDpNA Substrate at 37 °C for 4 h. The absorbance was measured at 405 nm. Caspase-9 activity was measured using luminescent assay method. The samples were mixed with the aliquot of Caspase-Glo^®^ 9 Reagent and incubated for 3 h at room temperature. The luminescence was measured in a plate-reading luminometer as directed by the luminometer manufacturer.

### 4.5. ROS Measurement

Production of ROS was monitored fluorimetrically 2 h after reoxygenation, using the fluorescent probe 2′-7′-dichlorofluorescein diacetate (DCFH-DA, Sigma). Samples were collected, rinsed with PBS, and incubated with 10 μM DCFH-DA at 37 °C for 30 min. Fluorescence was observed using an Axiovert 40 fluorescence microscope (Zeiss, Oberkochen, Germany). For the quantitative assay, the fluorescence intensity was measured by a microplate reader at an excitation wavelength of 485 nm and emission wavelength of 525 nm. Protein analysis was performed in accordance with the procedure described previously [47].

### 4.6. Determination of Intracellular Calcium Concentration

The intracellular Calcium concentration was detected by Fluo-3 AM (Beyotime Institute of Biotechnology, Haimen, China), following the manufacturer’s instructions. Briefly, cells were trypsinized, washed with PBS and incubated with Fluo-3 AM solution (5 μM) for 30 min at 37 °C. The fluorescence intensity of Fluo-3/AM-loaded cells was detected by a flow cytometer at 488 nm excitation and 525 nm emission wavelengths. Results were expressed as a percentage of vehicle-treated cells.

### 4.7. Measurement of Mitochondrial Membrane Potential

The mitochondrial membrane potential (MMP) was measured using a mitochondrial membrane potential assay kit with JC-1 (Beyotime Institute of Biotechnology, Haimen, China) according to the manufacturer’s protocol. Cells were trypsinized and washed with PBS, and then 0.5 mL DMEM and 0.5 mL JC-1-fluorescent dye were added to cells. After incubation at 37 °C for 20 min, the cells were centrifuged at 600 *g* for 3 min and the supernatant was removed. For flow cytometer analysis, cells were resuspended in JC-1 buffer. The monomeric form was detected at 490 nm excitation and 530 nm emission while the dimeric form was detected at 525 nm excitation and 590 nm emission. The results were expressed as the ratio of red to green fluorescence. 

### 4.8. Western Blot

At the end of treatment, cells were rinsed in PBS and lysed in RIPA lysis buffer. Twenty micrograms of protein were loaded into each lane, separated by 10% sodium dodecyl sulfate-polyacrylamide gel electrophoresis (SDS-PAGE) and transferred to nitrocellulose membranes (Pall Corporation, New York, NY, USA) in Tris-glycine buffer (48 mM Tris, 39 mM glycine, pH 9.2) containing 20% methanol. The membranes were blocked with skimmed milk for 1 h, washed in Tris buffered saline containing 0.1% Tween-20 (TBST) and incubated overnight with the primary antibodies (Bcl-2, 1:500 dilution; Bax, 1:1000 dilution; Akt, 1:5000 dilution; phospho-Akt, 1:2000 dilution; GSK-3β, 1:1000 dilution; phospho-GSK-3β (Ser9), 1:1000 dilution and GAPDH, 1:10,000 dilution). After washing three times with TBST, nitrocellulose membranes were incubated for 1 h at room temperature with horseradish peroxidase-conjugated goat anti-rabbit (Santa Cruz, 1:5000 dilution) or anti-mouse IgG (Santa Cruz, 1:5000 dilution). Bands were visualized using the SuperSignal West Pico Chemiluminescent Substrate Trial Kit (Pierce, Rockford, IL, USA). Images were taken using the ChemiDoc XRS system with Quantity One software (Bio-Rad, Richmond, CA, USA).

### 4.9. Statistical Analysis

All values are expressed as the mean ± standard deviation (SD) of at least three independent preparations. Differences among the groups were compared using one-way analysis of variance (ANOVA) analysis followed by a Tukey post-hoc test. A difference with *p* < 0.05 was considered statistically significant.

## 5. Conclusions

**Figure 6:**
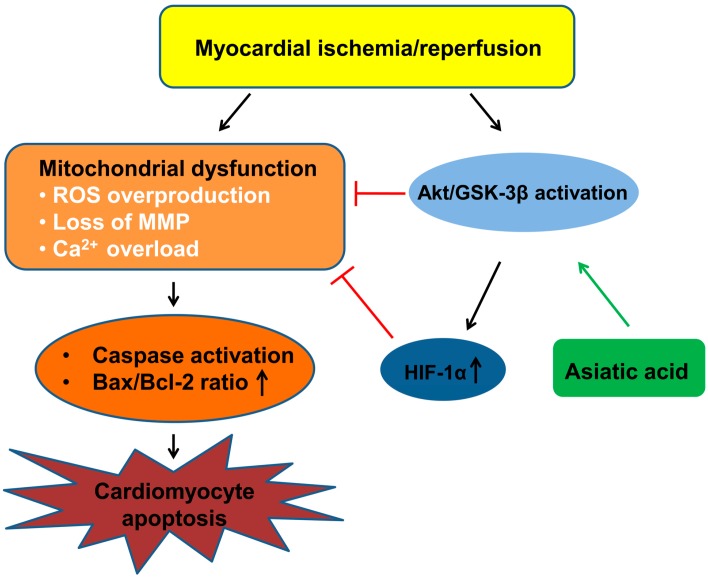
A schematic diagram of the mechanism by which asiatic acid prevents cardiomyocyte apoptosis. Ischemia/reperfusion induces mitochondrial dysfunction and triggers cardiomyocyte apoptosis. Asiatic acid activates the Akt/GSK-3β signal pathway and increases HIF-1α levels, thus suppressing the mitochondrial-mediated apoptosis.

In summary, the activation of Akt/GSK-3β mediated by asiatic acid offers protection to cardiomyocytes by promoting HIF-1α expression, which leads to the inhibition of ROS production, maintenance of MMP and attenuation of calcium overload, thus suppressing the mitochondrial-mediated apoptosis in hypoxic cardiomyocytes (Figure 6). Linking the AKT/GSK-3β pathway and HIF-1α in myocardial ischemia/reperfusion injury advances our understanding of the molecular mechanisms underlying the cardioprotection of asiatic acid.
